# Serum Metabolomic Signatures Indicate Oxidative Membrane Lipid Remodeling in β-Thalassemia

**DOI:** 10.3390/metabo16030170

**Published:** 2026-03-05

**Authors:** Alexandros Makis, Eleftheria Hatzimichael, Theodoros Palianopoulos, Dimitra Papagiannaki, Eleni Kapsali, Evangelos Gikas, Vasilios Sakkas

**Affiliations:** 1 Department of Pediatrics, University Hospital of Ioannina, Faculty of Medicine, University of Ioannina, 45500 Ioannina, Greece; 2 Department of Hematology, University Hospital of Ioannina, Faculty of Medicine, University of Ioannina, 45500 Ioannina, Greece; 3 Laboratory of Analytical Chemistry, Department of Chemistry, University of Ioannina, 45500 Ioannina, Greece; 4 Laboratory of Pharmaceutical Analysis, School of Pharmacy, University of Athens, 15744 Athens, Greece

**Keywords:** β-thalassemia, serum metabolomics, lysophosphatidylcholine, phospholipase A_2_, arachidonic acid, iron overload, bile acids

## Abstract

**Background/Objectives**: Oxidative stress and iron overload remodel erythrocyte membranes in β-thalassemia, but their systemic metabolic correlates are not well defined. We applied untargeted metabolomics to identify serum biomarkers reflecting these pathophysiological processes. **Methods**: Thirty-one adults with β-thalassemia [18 transfusion-dependent (TDT), 13 non-transfusion-dependent (NTD)] and 8 age/sex-matched healthy controls were studied. Fasting serum was profiled using untargeted UHPLC–Orbitrap MS. Multivariate modeling (SIMCA-P) and FDR-controlled univariate statistics identified discriminant features, followed by pathway enrichment analysis. Associations with clinical variables (chelation regimen, ferritin, cardiac MRI T2*, and liver iron concentration) were examined. **Results**: A total of 183 metabolites were detected; versus controls, 124 were decreased, 54 increased, and 5 remained unchanged in patients. Key discriminants included lysophosphatidylcholines (LysoPC 18:1, 18:3), polyunsaturated fatty acid (PUFA)-bearing phosphatidylcholines (PC 20:4/18:0, PC 18:0/20:4), conjugated bile acids (glycocholic acid, glycochenodeoxycholic acid, and glycoursodeoxycholic acid), and bilirubin. Pathway analysis revealed significant enrichment (FDR-corrected) in linoleic acid metabolism (q = 0.024, impact = 1.000) and arachidonic acid metabolism (q = 0.022, impact = 0.433), with supportive nominal signals from glycerophospholipid (impact = 0.401) and porphyrin/heme (impact = 0.242) pathways. No significant metabolic differences were observed between TD and NTD patients. **Conclusions**: β-thalassemia serum metabolomics reflects oxidative membrane lipid remodeling with a prominent PLA_2_/LysoPC–arachidonic axis and evidence of heme turnover and altered bile-acid signaling. These data propose a practical biomarker panel-LysoPCs, arachidonic acid-enriched PCs, and conjugated bile acids-warranting targeted validation alongside conventional clinical parameters for disease monitoring and therapeutic assessment.

## 1. Introduction

β-thalassemia is an autosomal-recessive hemoglobinopathy caused by deficient β-globin synthesis. Homozygous or compound heterozygous mutations produce transfusion-dependent (TD) disease with lifelong anemia, whereas milder genotypes may be non-transfusion-dependent (NTD) with intermittent transfusion needs. Excess unpaired α-chains form hemichromes that catalyze reactive oxygen species, driving lipid/protein oxidation, membrane destabilization, apoptosis of erythroblasts, and ineffective erythropoiesis; peripheral hemolysis further aggravates anemia [1,2]. Beyond hematologic manifestations, chronic hemolysis, endothelial adhesion, inflammation, oxidative stress, and iron overload contribute to multisystem—especially cardiovascular—injury [3,4].

From a genetic and epidemiological perspective, β-thalassemia is an autosomal-recessive disorder caused by pathogenic variants in the HBB gene located within the β-globin gene cluster on chromosome 11 (11p15.5); accordingly, inheritance and population allele frequency are not expected to differ by sex. Age primarily influences clinical expression, cumulative complications, and survival rather than mutation prevalence. Advances in transfusion regimens, iron chelation, and multidisciplinary follow-up have progressively improved survival, resulting in an expanding adult and aging β-thalassemia population in settings with sustained access to modern care [1,2]. Consequently, wide age distributions and occasional sex imbalances observed in contemporary single-center cohorts mainly reflect survivor effects, referral patterns, and sampling variability rather than biologically meaningful differences in disease frequency.

Metabolomics, by quantifying small-molecule intermediates and end products [5,6], has begun to delineate the systemic biochemical footprint of these processes across human cohorts and experimental models, but findings remain heterogeneous with respect to matrix, platform, and clinical context. In patients, NMR/GC–MS/LC–MS investigations have reported broad changes spanning organic acids, amino acids, redox/heme catabolites, and lipid classes. For example, urine metabolomics in transfusion-dependent β-thalassemia under deferasirox therapy revealed treatment-linked shifts in small-molecule pathways (organic acids and energy metabolites) [7]. Targeted and untargeted approaches in patient sera have proposed candidate markers of iron load and oxidative damage [8], and untargeted serum profiling has demonstrated significant deviations from healthy controls—including pathways related to carbohydrate, fatty acid, and glycerophospholipid metabolism [9]. Importantly, metabolic re-alignment toward a healthier profile has been observed in hydroxyurea-treated β-thalassemia children, underscoring therapy-responsive biochemical plasticity [10]. Beyond blood and urine, fetal chorionic villi from β-thalassemia pregnancies showed oxidative-stress and inflammatory signatures by metabolomics [11]. At the tissue and immune-cell level, recent multi-omic work in β-thalassemia and sickle models demonstrates pronounced metabolic/proteomic divergence in spleen and liver and between circulating monocytes vs. tissue macrophages, highlighting organ- and cell-type–specific remodeling that plausibly echoes in the circulating metabolome [12,13]. In contemporary patient cohorts, cellular/biochemical heterogeneity has been linked to phenotypic diversity among transfusion-dependent β-thalassemia, reinforcing the need for systemic readouts that integrate red-cell, hepatic, and inflammatory axes [14]. Finally, integrative microbiome–metabolome analyses have implicated gut–liver crosstalk—in particular, taxa influencing bilirubin/stercobilin turnover—as potential modifiers of anemia and iron burden [15].

In β-thalassemia, iron overload and ongoing hemolysis amplify oxidative stress, a relationship captured by metabolomic readouts of iron load and oxidative damage in patient cohorts [8] and by platform-consistent alterations spanning energy and lipid pathways in urine, serum, and fetal tissues [7,9,11,14]. At the red-cell interface, oxidative injury to membrane phospholipids promotes phospholipase A_2_–mediated remodeling, increasing lysophosphatidylcholines (LysoPCs) and mobilizing polyunsaturated fatty acids for eicosanoid/oxylipin biosynthesis—changes coherent with untargeted serum metabolomics that highlights disruption of glycerophospholipid metabolism and therapy-responsive metabolic plasticity [9,10]. Concomitantly, iron overload suppresses the farnesoid X receptor (FXR) in mouse and human hepatocytes, thereby dysregulating bile-acid homeostasis and potentiating iron-induced hepatotoxicity [16]. This hepatocellular axis likely intersects with gut–liver crosstalk, as microbiome–metabolome analyses in β-thalassemia implicate taxa modulating bilirubin/stercobilin turnover and secondary bile-acid formation as potential modifiers of anemia and iron burden [15]. Together, these observations support a unified model wherein iron-driven oxidative stress couples erythrocyte membrane lipid remodeling with bile-acid dysregulation, motivating our serum metabolomics focus on PLA_2_/LysoPC–arachidonate and conjugated bile-acid signatures.

Here, we conducted untargeted UHPLC–Orbitrap MS metabolomics of fasting serum from patients with β-thalassemia and age/sex-matched healthy controls, integrating multivariate and FDR-controlled univariate statistics, pathway enrichment, and quantitative associations with ferritin, liver iron concentration, and cardiac T2*. Our explicit novelty relative to prior reports is threefold: (1) a serum-centered, untargeted LC–HRMS analysis that jointly interrogates PLA_2_/LysoPC–arachidonic remodeling and bile-acid dysregulation within the same cohort; (2) framing these axes into a concise, biomarker panel (LysoPCs, AA-enriched PCs, conjugated bile acids) suitable for targeted validation; and (3) evaluating clinical correlations with standardized iron metrics (ferritin, LIC, cardiac T2*), thereby enhancing translational relevance and paving the way for longitudinal monitoring. By mapping these metabolic derangements, we aim to identify actionable biomarkers and therapeutic targets, facilitating the transformation from a hemoglobin-centered view to a systems-level framework that can guide targeted validation and future therapeutic strategies in β-thalassemia.

## 2. Materials and Methods

The study included 31 patients with β-thalassemia living in the region of Northwestern Greece and followed at the pediatric and adult thalassemia Units at the University Hospital of Ioannina. The diagnosis was based on clinical, hematological, biosynthetic, and genetic studies. Medical files of the patients were retrieved, and detailed data were recorded and analyzed. Other parameters, such as serum ferritin, cardiac MRI T2*, MRI liver iron concentration (LIC), and chelation regimen, were enrolled in the study. The demographic, laboratory, and clinical data are shown in Table 1. Fourteen patients were male, and the age range was 12–67 years, with a median of 36 years. Eighteen had TD β-thalassemia, and 13 had NTD. Eight apparently healthy individuals, matched to the patient cohort for age and sex distribution, served as controls (age range 13–62 years; median 35.5 years, 4 male). All participants provided written informed consent, and the study was conducted in accordance with the Declaration of Helsinki.

Chelation regimen at sampling was recorded as none, deferiprone (DFP), deferasirox (DFX), deferoxamine (DFO), or combination therapy (e.g., DFP + DFX or DFP + DFO). Decisions to initiate or withhold chelation followed standard practice thresholds integrating serum ferritin trends, LIC, and cardiac T2* [17,18]. DFO is a parenteral hexadentate chelator with a short half-life; the iron–DFO complex (ferrioxamine) is mainly eliminated renally, and DFO remains an option for severe iron overload or when oral agents are unsuitable. DFP is an oral bidentate chelator (typically three times a day) that promotes urinary iron excretion and is well recognized for efficacy in myocardial siderosis; idiosyncratic agranulocytosis mandates blood count monitoring. DFX is an oral tridentate, once-daily chelator with predominantly fecal iron excretion and proven efficacy for hepatic iron reduction/maintenance. Combination therapy (most commonly DFP + DFO; less often DFX with another agent) is used in difficult-to-treat overload or cardiac siderosis and can accelerate iron removal [17,18]. Because these agents differ in coordination chemistry, dosing, and excretory pathways (renal vs. biliary/fecal), chelation may modulate systemic metabolites; accordingly, we recorded the regimen per subject (Table 1) and considered chelation as a covariate in sensitivity analyses.

During recruitment, patients with evidence of other chronic illnesses unrelated to thalassemia (such as diabetes mellitus, autoimmune disorders, chronic kidney disease, or active malignancy) were excluded to minimize confounding metabolic alterations. The final study cohort comprised the 31 patients detailed in Table 1. Peripheral blood was drawn after an overnight fast and, for transfused patients, prior to the scheduled transfusion to minimize acute post-transfusion effects. Samples were allowed to clot, centrifuged to obtain serum, aliquoted to avoid repeated freeze–thaw, and stored at −80 °C until analysis. All were performed on ice or at 4 °C (except vortexing) to limit ex vivo metabolic change and oxidation. Sterile pipettes and tips were used, and appropriate precautions were taken during the performance of assays (using a laminar flow instrument, Telstar/Bio II A, Telstar, Terrassa, Spain) in a cell culture room) to protect samples from subsequent environmental contamination. Acetonitrile (ACN), methanol (MeOH), and formic acid (FA) were obtained from Fluka/Riedel-de Haën (Buchs, Switzerland). Millipore’s Milli-Q Plus water purification system (Milford, MA, USA) was utilized to create high-quality purified water.

**Figure 1:**
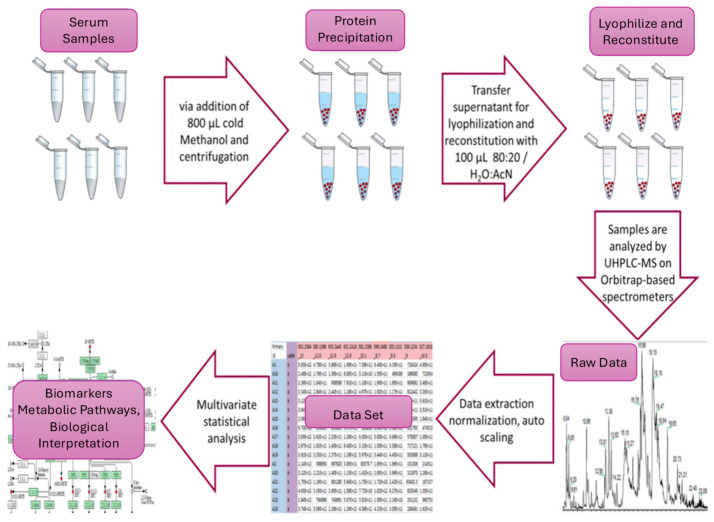
Untargeted serum metabolomics workflow in β-thalassemia.

Sample pretreatment. The serum samples were thawed on ice at 4 °C for 30 to 60 min, depending on the quantity of the available samples. Consequently, an aliquot of 200 μL was drawn from each sample, and 800 μL of MeOH was added, followed by 15 s of vortexing. Afterward, the samples were centrifuged at 12,000× *g* for 15 min at 4 °C to separate the protein precipitate. The supernatant was transferred to a 1.5 mL Eppendorf tube, vacuum-evaporated (GeneVac). HT-4X EZ-2 series evaporator Lyospeed ENABLED (Genevac. Ltd., Ipswich, UK) at 50 °C, and stored (−80 °C) until analysis. For HRMS analysis, the samples were reconstituted (100 μL 80:20 H_2_O:ACN (*v*:*v*)), vortexed for 30 s, and transferred to 200 μL inserts in appropriate screw-capped autosampler vials. Throughout these processes, the samples were kept on ice before each centrifugation. A pooled quality control (QC) sample was constructed by aliquoting 10 μL from each sample before evaporation. The combined mixture underwent the pretreatment described, adjusted for the volume. Sample handling and analysis are depicted as a workflow in Figure 1.

Samples were analyzed using an LC-MSn system comprised of an ESI-LTQ-Orbitrap Discovery XL mass spectrometer connected to an Accela UHPLC system (Thermo Scientific, Bremen, Germany). The UHPLC setup included an autosampler, vacuum degasser, binary pump, and a column with controlled temperature. Analysis was conducted using a Waters Corp. ACQUITY UPLC BEH C18 column (100 × 2.1 mm, 1.7 μm particle ID, Waters Corporation, Milford, MA, USA). The DDA analysis mode was selected, and the fragmentation was done in the linear ion trap part.

Before conducting the multivariate statistical analysis, the data underwent preprocessing using several tools. Xcalibur^®^ from Thermo Fisher Scientific (San Jose, CA, USA), MZmine 2.10 available at mzmine.sourceforge.net. Microsoft Excel and the web-based MetaboAnalyst 6.0 suite [19] were utilized. Initially, Xcalibur^®^ converted the instrument’s native data files (.raw) to the cdf data format (.cdf) through the Xcalibur^®^ Xconvert program. Subsequently, MZmine 2.10 processed the data, employing baseline correction, peak detection, deconvolution, deisotoping, alignment, and gap-filling procedures. The resulting peak list, displaying accurate mass-tR versuadds intensity, was exported as a .csv file to Microsoft Excel and further manipulated using commands such as CONCATENATE, ROUND, and TRANSPOSE for appropriate adjustments.

For the statistical analyses, both univariate and multivariate statistics have been employed. The online version of Metaboanalyst 6.0 has been used for the univariate analyses, whereas for their multivariate counterparts, SIMCA P+ 10.5 (Umetrics, Umea, Sweden) and EZinfo 2.0 (Umetrics, Umea, Sweden) were also employed. Briefly, PCA was employed for revealing trends and locating putative outliers, whereas PLSDA was used for deciphering the clustering of the thalassemia vs. normal control subjects. The results were also verified employing univariate statistics (*t*-test and fold-change analyses). The methodology has been included in the Supplementary Material.

## 3. Results

### 3.1. Global Serum Metabolome and Data Structure

Untargeted UHPLC–Orbitrap MS detected 183 serum features across patients and controls. Based on combined multivariate and univariate analyses, 124 features were decreased, 54 were increased, and 5 were unchanged in patients versus controls.

**Figure 2:**
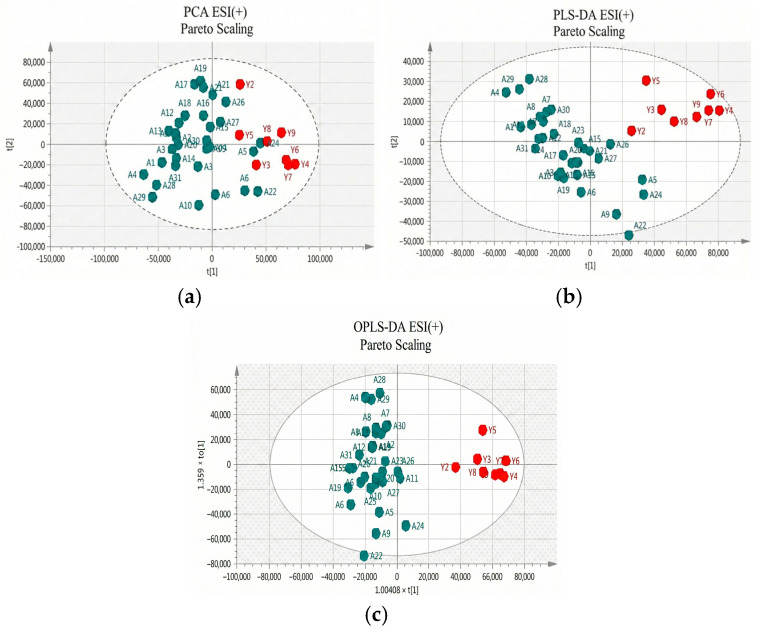
(**a**) PCA score plot in Pareto scaling with R^2^ = 0.610, Q^2^ = 0.364, and 5 principal components; (**b**) PLS-DA score plot in Pareto scaling with R^2^ = 0.853, Q^2^ = 0.601, and 2 principal components; and (**c**) OPLS-DA score plot in Pareto scaling with R^2^ = 0.913, Q^2^ = 0.715, and 3 principal components for UHPLC-MS serum analysis in positive ionization ESI (+) between controls (red) and patients (green).

**Figure 3:**
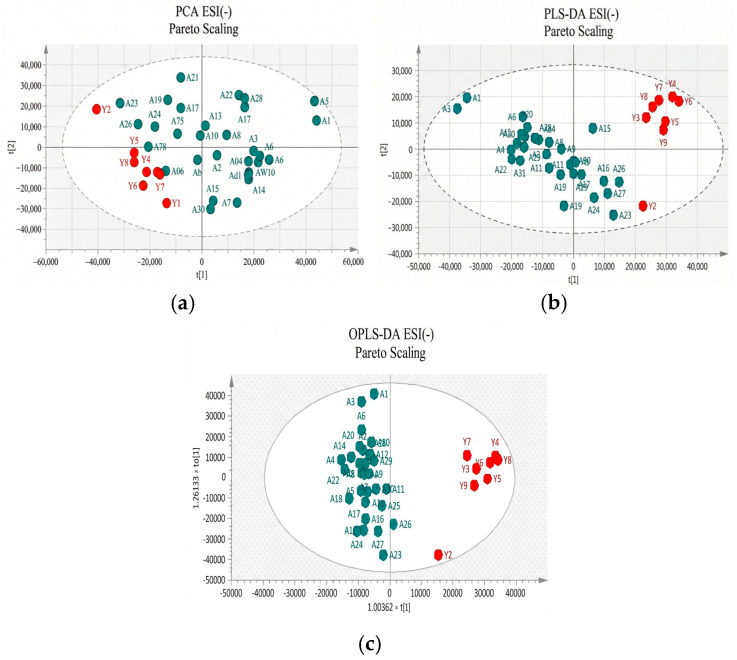
(**a**) PCA score plot in Pareto scaling with R^2^ = 0.302, Q^2^ = 0.141, and 2 principal components; (**b**) PLS-DA score plot in Pareto scaling with R^2^ = 0.799, Q^2^ = 0.447, and 2 principal components; and (**c**) OPLS-DA score plot in Pareto scaling with R^2^ = 0.924, Q^2^ = 0.514, and 4 principal components for UHPLC-MS serum analysis in negative ionization ESI (−) between controls (red) and patients (green).

Two approaches were adopted for data scaling: UV (auto-scaling) and Pareto scaling for both negative and positive ion modes. UV scaling equalizes the importance of all metabolites but amplifies baseline noise. Conversely, Pareto scaling retains the original measurements to a greater extent but is responsive to significant changes [20]. As depicted in Figure 2 and Figure 3 for positive and negative ion modes, respectively, the samples showed close clustering in PCA score plots. However, the distinction between the two groups was not deemed satisfactory, and therefore supervised methods, such as PLS-DA or OPLS-DA, were employed.

Both methods were able to distinguish between the two sets (controls versus patients), displaying distinctive clustering that suggested varying metabolic profiles in both modes, both for UV and Pareto scaling. However, in these supervised models, where users categorize samples into pre-established groups, there is the risk of creating biased clustering or overfitting. Hence, permutation testing involving 100 permutations was used to evaluate the significance of the classification, utilizing the prediction accuracy during training as a statistical test. The results of the permutation test indicated that UV scaling was overfit to the data since R2 and Q2 values derived from the permuted data exceeded the original values on the validation plot. This indicates that relying on the model using UV scaling might not be dependable for subsequent statistical analysis, as it fails to precisely represent the inherent patterns within the data.

### 3.2. Feature Selection and Identification

**Figure 4:**
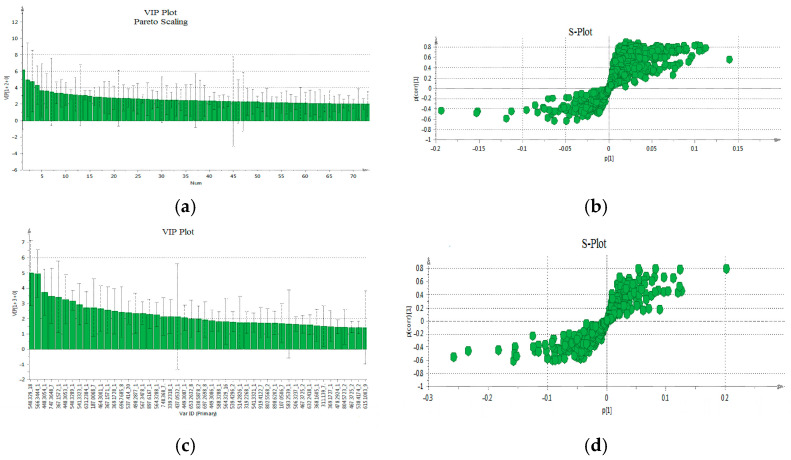
The VIP-plot and S-plot in positive (**a**,**b**) and negative (**c**,**d**). (**a**) VIP-plot and (**b**) S-plot of OPLS-DA. In the VIP-plot, the variables are ranked from the most statistically significant to the least. In the S-plot, the features positioned at the extremes, farthest from the central cluster, display substantial variation and consistency, thereby holding significant importance when searching for potential biomarkers (characteristics in the lower left quadrant belong to the patients, while those in the upper right quadrant belong to the healthy individuals).

The VIP-plot and S-plot in positive (Figure 4a,b) and negative (Figure 4c,d) mode were computed to pinpoint potentially crucial variables that contribute to the distinct clustering observed in the OPLS. VIP values (>1.0) were utilized to pinpoint the metabolites responsible for distinguishing between the two groups.

**Figure 5:**
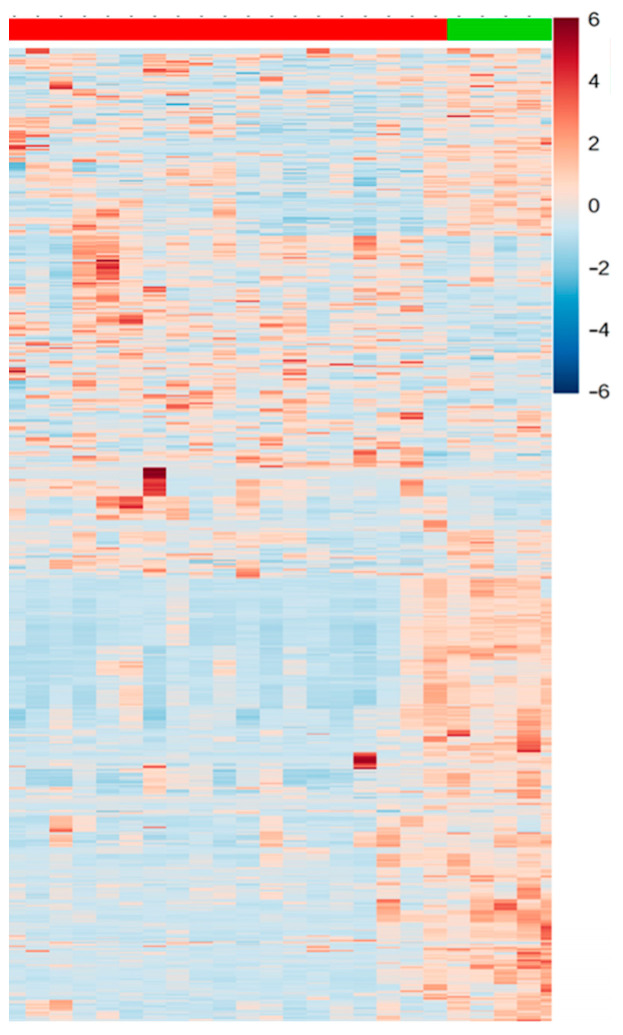
Heatmap representation of significant differences between patients (red) and controls (green).

Additionally, to validate the outcomes related to the selection of VIPs from both modes (positive and negative), univariate data analysis was conducted using *t*-tests, FC monitoring, and their amalgamation in the form of a heatmap plot (Figure 5).

By merging the findings from both univariate and multivariate analyses, a compilation of features that differentiate the two groups was assembled for structural identification. The identification strategy involved comparing the selected features with online databases based on mass accuracy, isotopic patterns, RDBeq information, and potential low-energy or in-source fragmentation data for assessment. As a result, 20 metabolites (Table 2) were recognized via Orbitrap-based analysis.

### 3.3. Metabolic Pathway Analysis

**Figure 6:**
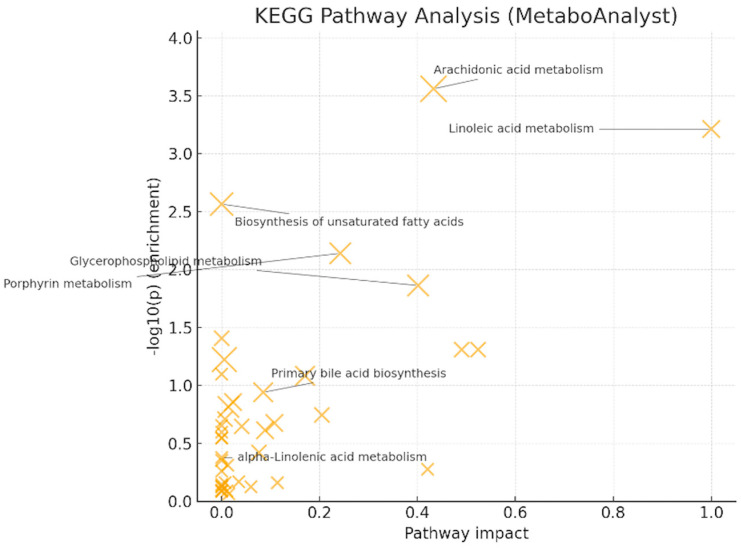
KEGG pathway analysis (MetaboAnalyst 6.0) [–log10(*p*) vs. pathway impact; bubble size = hits. Significant after FDR: linoleic acid (q = 0.0245, impact = 1.000) and arachidonic acid (q = 0.0220, impact = 0.433). Glycerophospholipid and porphyrin are only nominal].

Pathway analysis (MetaboAnalyst/KEGG) [19] showed significant enrichment for arachidonic acid metabolism (q = 0.022, impact = 0.433) and linoleic acid metabolism (q = 0.024, impact = 1.000) (Figure 6). Glycerophospholipid metabolism (q = 0.220, impact = 0.401) and porphyrin metabolism (q = 0.145, impact = 0.242) reached nominal significance but did not survive FDR correction. These patterns, together with LysoPC up-regulation at the feature level, support PUFA (polyunsaturated fatty acid)- and membrane-lipid-centered remodeling under oxidative stress, with eicosanoid precursor flux as a plausible downstream consequence. Primary bile-acid biosynthesis and α-linolenic acid metabolism were not significant after correction in this dataset.

### 3.4. Subgroup and Clinical Correlations

Within the β-thalassemia cohort, no significant differences were observed between TDT and NTDT groups in global multivariate structure or in individual metabolites. This lack of separation persisted after considering demographic, clinical, and laboratory covariates provided in Table 1.

### 3.5. Study Limitations

Limitations of the study include modest sample size; class imbalance (31 patients vs. 8 controls); wide age range; potential confounding by transfusion timing, chelation, diet, and microbiota being not fully controlled; and the inherent constraints of untargeted annotation. Larger cohorts are needed for confirmation. Furthermore, our cross-sectional design may underpower subgroup contrasts (TDT vs. NTDT) and cannot resolve causality. We did not quantify PLA_2_ activities, eicosanoids, FXR/TGR5 signaling, FGF19, or gut microbial composition; therefore, mechanistic interpretations involving these pathways should be viewed as exploratory. Findings should be viewed as hypothesis-generating and require targeted validation in larger, multi-center cohorts with independent replication.

## 4. Discussion

The present study utilized untargeted metabolomic analysis to identify a set of significantly up-regulated metabolites that cluster into two biologically coherent groups: (A) phosphatidylcholine (PC)–related species along the glycerophospholipid axis and (B) conjugated bile acids belonging to primary/secondary bile-acid biosynthesis. Together, these findings support a unifying model in β-thalassemia whereby oxidative membrane remodeling of erythrocytes and iron-related hepatobiliary signaling leave distinct, measurable footprints in the serum metabolome.

A. Phosphatidylcholine biosynthesis and remodeling

Several phosphatidylcholines (PCs) and lysophosphatidylcholines (LysoPCs) were significantly elevated in β-thalassemia patients compared to healthy controls.

*Phosphatidylcholines.* PC(O-14:0/2:0) or 1-tetradecyl-2-acetyl-sn-glycero-3-phosphocholine PC(0:0/18:1(9E) or 2-oleoyl-sn-glycero-3-phosphocholine; PC(17:2(9Z,12Z)/19:0) or 1,2-diacyl-sn-glycero-3-phosphocholine; PC(20:4(5Z,8Z,11Z,14Z)/18:0); and PC(18:0/20:4(8Z,11Z,14Z,17Z)) are PCs (glycerophospholipids) in which a phosphorylcholine moiety occupies a glycerol substitution site which are intermediates in the metabolic pathway of PCs. PCs and sphingomyelin are the dominant phospholipids of the erythrocyte outer membrane layer, whereas phosphatidylethanolamine and phosphatidylserine (PS) reside largely on the inner layer. This transbilayer asymmetry—maintained by flippases/floppases/scramblases—is essential for RBC deformability and for evading macrophage recognition [21]. In β-thalassemia, excess α-globin chains, labile iron, and elevated intracellular Ca^2+^ promote lipid peroxidation and scramblase activation, causing PS externalization that accelerates splenic clearance and contributes to vascular/thrombotic complications [22]. Furthermore, phosphocholine and phosphatidylcholine catabolism play a key role in ATP production and terminal erythropoiesis, and the deregulation of this process may contribute to ineffective erythropoiesis in thalassemia [23].

*Lysophosphatidylcholines (LysoPCs).* LysoPC(18:1(11Z)) and LysoPC(18:3(9Z,12Z,15Z)) are monoacyl-glycerophospholipids bearing a phosphorylcholine headgroup. LPCs arise primarily from PC hydrolysis by lipoprotein-associated phospholipase A_2_ (Lp-PLA_2_) and from lecithin:cholesterol acyltransferase (LCAT) during cholesterol esterification. Beyond their structural role, LysoPCs function as bioactive lipids: they engage lysophospholipid-responsive pathways, generally upregulating genes involved in cholesterol biosynthesis while suppressing programs of hepatic fatty-acid oxidation, and at elevated concentrations they provoke endothelial dysfunction and oxidative stress. Excess LysoPCs production can reflect increased PLA_2_ flux, including Lp-PLA_2_ overexpression/activation [24]. Notably, β-thalassemia patients exhibit elevated circulating Lp-PLA_2_ activity [25], plausibly driven by monocyte/macrophage activation and heightened lipoprotein/erythrocyte oxidative stress. Together with our data, these observations support a mechanistic model in which erythroid and systemic redox imbalance expands the pool of oxidized PCs; PLA_2_ activities (including Lp-PLA_2_) cleave these substrates to generate LysoPCs that accumulate in the circulation and, in turn, amplify endothelial and inflammatory signaling. Because our untargeted approach does not quantify enzyme activities or downstream oxylipins, direct measurements of Lp-PLA_2_/sPLA_2_/cPLA_2_ activities and targeted eicosanoid profiling will be required to test this mechanism.

*Arachidonic acid (AA).* We detected enrichment of arachidonic-acid–linked pathways, with AA-related features among the top discriminants. AA is a PUFA esterified predominantly in phosphatidylethanolamine, phosphatidylcholine, and phosphatidylinositol pools of cellular membranes. Upon activation, PLA_2_ hydrolyzes the sn-2 ester bond to liberate free AA, which is then converted by cyclooxygenases (COX-1/2), lipoxygenases (5-/12-/15-LOX), and cytochrome P450 epoxygenases into eicosanoids—potent autacoids governing inflammation, vascular tone, platelet function, and leukocyte trafficking [26].

In our cohort, the up-regulation of arachidonic-acid methyl ester and related signals indicates accelerated PUFA turnover and a shift toward pro-inflammatory oxylipin biosynthesis, consistent with elevated oxidative stress and sterile inflammation. These findings align with prior untargeted metabolomics in β-thalassemia that reported broad perturbations in lipid and energy pathways and a chronic pro-inflammatory milieu [9]. In thalassemia patients, chronic hemolysis and iron-driven reactive oxygen species (ROS) production may both increase PLA_2_-dependent AA liberation and promote non-enzymatic PUFA peroxidation, yielding non-classic eicosanoids, such as isoprostanes, that further amplify vascular inflammation [25,27]. Although we did not measure individual eicosanoids or PLA_2_ activities, the combined signatures—elevated LysoPCs, perturbation of PUFA-rich PCs, and pathway enrichment—are compatible with increased AA turnover. Confirmation will require targeted oxylipin MS and enzymatic assays.

B. Bile acid biosynthesis

We observed up-regulation of conjugated primary bile acids—glycocholic acid (GCA) and glycolchenodeoxyglycocholic acid (GCDCA),—of the C27 intermediate (25R)-3α,7α-dihydroxy-5β-cholestan-27-oyl taurine alongside the secondary species glycoursodeoxycholic acid (GUDCA). Primary bile acids are synthesized from cholesterol in hepatocytes and conjugated to glycine/taurine before biliary secretion; most are reabsorbed in the ileum, but a fraction undergoes microbial deconjugation and 7α-dehydroxylation/epimerization to form secondary bile acids, e.g., ursodeoxycholic acid (UDCA), GUDCA, and deoxycholic acid (DCA) [28]. The elevation of bilirubin and primary bile acids in our patients suggests coupled effects of hemolysis-driven heme turnover and hepatic stress. The increased levels of bile acids may have several systemic effects, such as deterioration of hemolysis [29].

Functionally, bile acids can influence erythrocyte stability and systemic metabolism via the nuclear receptor FXR (farnesoid X receptor) and TGR5/GPBAR1 (Takeda G protein-coupled receptor 5/G protein-coupled bile acid receptor 1) receptor—pathways that regulate FGF19 (fibroblast growth factor 19) signaling [13]. We did not assess FXR/TGR5 activity, FGF19, or hepatic transcriptomics in this study; therefore, any link to receptor signaling should be considered hypothesis-generating. Prior work has reported that iron overload can suppress hepatocellular FXR and perturb bile-acid homeostasis [13]; our detection of a taurine-conjugated C27 intermediate is compatible with, but does not establish, such dysregulation in our cohort.

The presence of GUDCA could reflect microbiome-dependent epimerization of chenodeoxycholic acid followed by hepatic reconjugation [16]; however, we did not profile the gut microbiome, and hepatic and biliary handling may also contribute. Accordingly, any inference about microbiome involvement remains speculative and warrants integrated microbiome–metabolome studies.

## 5. Conclusions

Untargeted UHPLC–Orbitrap MS metabolomics of β-thalassemia serum samples revealed a dual metabolic signature: (i) disrupted glycerophospholipid metabolism with elevated PC/LysoPC species and (ii) altered bile acid biosynthesis marked by increased conjugated bile acids and a taurine-linked C27 intermediate. These findings suggest that oxidative stress from iron overload and hemolysis drives membrane remodeling and inflammation, while hepatobiliary and gut-derived signals reshape the bile acid pool. Notably, these metabolic alterations were consistent across TD and NTD patients. The results nominate potential biomarker candidates (LysoPCs, AA-enriched PCs, conjugated bile acids) and generate hypotheses regarding pathways (oxidative stress, PLA_2_, bile-acid signaling) for future validation. Larger longitudinal studies integrating metabolomics with clinical outcomes are needed for biomarker validation and clinical translation.

## Figures and Tables

**Table 1:** Demographic, clinical, and laboratory data from 31 thalassemia patients.

Patient Number	Age, Years	Gender	Type of Thalassemia	Ferritin (μg/L)	Iron Chelator	LIC (mg Fe/g Dry Weight)	Heart MRI T2* (ms)
1	18	F	NTD	1250	DFX	4.2	30.1
2	32	M	NTD	780	DFX	4.1	29.2
3	21	M	NTD	1406	DFX	4.9	30.4
4	49	M	NTD	460	DFO + DFP	3.1	21.1
5	13	F	NTD	777	DFP	3.9	28.8
6	62	M	NTD	377	DFX	3.9	38.6
7	43	F	NTD	2036	DFO + DFP	6.1	13.8
8	47	F	NTD	648	DFX	4.7	31.3
9	13	M	NTD	199	None	2.8	35.2
10	17	M	NTD	172	None	3.8	37.7
11	15	M	NTD	277	None	2.9	31.7
12	67	M	NTD	646	DFX	4.5	23.2
13	50	M	NTD	284	DFO + DFP	3.9	34.8
14	49	M	TD	230	DFO + DFP	2.8	26.8
15	12	F	TD	2167	DFX	6.2	23.1
16	13	F	TD	2740	DFX	5.4	23.7
17	66	F	TD	1469	DFX	4.4	19.1
18	25	F	TD	1020	DFX	5.6	19.5
19	34	F	TD	280	DFX	2.9	31.8
20	28	F	TD	5713	DFP	6.9	38.3
21	43	F	TD	801	DFX	4.9	31.0
22	39	F	TD	726	DFP	3.6	33.8
23	33	M	TD	479	DFP	3.8	29.4
24	28	M	TD	418	DFP	4.8	27.6
25	45	F	TD	701	DFX	4.9	36.0
26	63	F	TD	266	DFP	3.2	26.6
27	50	F	TD	1240	DFP	4.0	30.4
28	36	M	TD	1066	DFX	5.1	34.2
29	39	M	TD	266	DFX	3.3	37.2
30	36	F	TD	995	DFX	4.9	32.8
31	39	F	TD	296	DFX	3.0	36.9

LIC = liver iron concentration, DFP = deferiprone, DFX = deferasirox, DFO = deferoxamine.

**Table 2:** List of the most significant up-regulated metabolites in β-thalassemia patients in comparison to healthy controls.

*m*/*z* RT	VIP Value	*p* Value	Identification
496.3388_18.1	618.569	0.0021	PC(O-14:0/2:0) [U]
540.329_18.1	501.372	0.0072	(25R)-3alpha,7alpha…-oyl taurine
991.6709_18.1	497.706	0.0001	PC(0:0/18:1(9E))
566.3444_18.7	496.688	0.0015	unidentified
522.3544_18.6	480.667	0.0011	LysoPC(18:1(11Z))
786.6001_19.9	431.837	0.0037	PC(17:2(9Z,12Z)/19:0)
448.3054_13.4	373.599	0.0042	Glycoursodeoxycholic acid
633.2547_12.9	364.633	0.0014	BQ 518
448.3053_13.7	327.565	0.0011	unidentified
540.3289_17.6	318.967	0.0001	unidentified
585.2696_11	317.973	0.0001	Bilirubin
810.6_20	279.483	0.0122	PC(20:4(5Z,8Z,11Z,14Z)/18:0)
631.2384_13	272.556	0.0008	unidentified
464.3001_12	267.582	0.0021	Glycocholic acid
831.6408_10.1	266.528	0.0310	PC(18:0/20:4(8Z,11Z,14Z,17Z))
537.414_20.7	252.404	0.0001	unidentified
498.2877_14.4	236.346	0.0019	LysoPC(18:3(9Z,12Z,15Z))
567.3478_18.7	235.863	0.0009	unidentified
897.6167_13.4	230.278	0.0003	Chenodeoxyglycocholic acid
339.2318_19.7	214.343	0.0020	Arachidonic acid methyl ester

*m*/*z* = mass-to-charge ratio, RT = retention time, VIP = variable importance in projection, PC = phosphatidylcholine, LysoPC = lysophosphatidylcholine.

## Data Availability

Dataset available on request from the authors.

## Supplementary Materials

The following supporting information can be downloaded at https://www.mdpi.com/article/10.3390/metabo16030170/s1. Section S1: Instrumental Conditions [30]; Section S2: Feature Annotation; Section S3: Statistical Analyses.

## Abbreviations

The following abbreviations are used in this manuscript:

AA	Arachidonic acid
ATP	Adenosine triphosphate
BA	Bile acid (s)
COX	Cyclooxygenase
DCA	Deoxycholic acid
DFO	Deferoxamine
DFP	Deferiprone
DFX	Deferasirox
ESI	Electrospray ion source
FC	Fold change
FDR	False discovery rate
FGF19	Fibroblast growth factor 19
FTMS	Fourier transform mass spectrometry
FWHM	Full width at half maximum
FXR	Farnesoid X receptor
GC	Gas chromatography
GCA	Glycocholic acid
GCDCA	Glycolchenodeoxyglycocholic acid
GPBAR1	G protein-coupled bile acid receptor 1
GUDCA	Glycoursodeoxycholic acid
HRMS	High-resolution mass spectrometry
IQR	Interquartile range
KNN	k-nearest neighbor
LCAT	Lecithin:cholesterol acyltransferase
LIC	Liver iron concentration
LOOCV	Leave-one-out cross-validation
LOX	Lipoxygenases
Lp-PLA2	Lipoprotein-associated phospholipase A_2_
LTQ	Linear trap quadrupole
LysoPC	Lysophosphatidylcholine
MRI	Magnetic resonance imaging
MS	Mass spectrometry
NMR	Nuclear magnetic resonance
NTDT	Non-transfusion-dependent thalassemia
OPLS-DA	Orthogonal projection to latent structures–discriminant analysis
PC	Phosphatidylcholine
PCA	Principal Component Analysis
PLA	Phospholipase
PLS-DA	Partial least square–discriminant analysis
PS	Phosphatidylserine
PUFA	Polyunsaturated fatty acid
QC	Quality control
RBC	Red blood cell
ROS	Reactive oxygen species
SIMCA-P	Soft independent modeling of class analogy (Pro)
T2*	Myocardial T2* (cardiac MRI)
TDT	Transfusion-dependent thalassemia
TGR5/GP	Takeda G protein-coupled receptor 5/G protein-coupled bile acid receptor 1
UDCA	Ursodeoxycholic acid
UHPLC	Ultrahigh-performance liquid chromatography
USA	United States of America
UV	Unit variance
VIP	Variable importance in projection
