# Supplementary material for: Serum Metabolomic Signatures Indicate Oxidative Membrane Lipid Remodeling in β-Thalassemia
Source: Metabolites. 2026 Mar 5;16(3):170. doi: 10.3390/metabo16030170 (PMC13027618; doi:10.3390/metabo16030170)
Supplement: Supplementary file 1 [file metabolites-16-00170-s001.zip › metabolites-4120977-supplementary.pdf]

Supplementary Materials

Section S1. Instrumental Conditions

The gradient method spanned 30 min with specific solvent ratios at different time points. The column temperature was maintained at 40 °C and the autosampler tray temperature at 5 °C. The injection volume was 10 μL. In positive ion mode, the capillary temperature and voltage were set to 320 °C and 40 V, respectively; the sheath gas flow was 40 arb. units, aux gas flow 8 arb. units, spray voltage 3.30 kV, and tube lens voltage 120 V. In negative ion mode, the capillary temperature and voltage were set to 320 °C and −40 V, respectively; the sheath gas flow and aux gas flow were maintained at 40 and 8 arb. units, the spray voltage was 2.70 kV, and the tube lens voltage was −120 V. In both ion modes, analysis employed Fourier-transform mass spectrometry (FTMS) in full-scan mode over *m/z* 100–1000 at a resolution of 30,000 FWHM; spectra were acquired in centroid mode. Chromatographic conditions: mobile phase A, aqueous 0.1% formic acid (v/v); mobile phase B, ACN. Elution program: 0 min, A/B 95:5; 3 min, A/B 95:5; 21 min, A/B 5:95; 23 min, A/B 5:95; 24 min, A/B 95:5; 30 min, A/B 95:5. Column temperature was 40 °C; injection volume, 10 μL; flow rate, 400 μL/min; autosampler temperature, 5 °C [30].

Section S2. Feature Annotation

Of the detected features, only those with an annotation confidence level of 3a or 3b were reported, in accordance with the Szymanski ruleset. These correspond to features with an MS/MS spectrum showing a cosine similarity ≥0.8, or with a consistent *in silico* fragmentation pattern. All remaining annotations were assigned to confidence level 4 and were not granted metabolite status. No analytical standards were available; therefore, annotation confidence levels 1 or 2 (Metabolomics Standards Initiative) could not be assigned.

Section S3. Statistical Analyses

Statistical analysis was performed using the MetaboAnalyst platform (online implementation). Data preprocessing included: (a) imputation of missing values using the k-nearest neighbor (KNN) algorithm; (b) noise filtering by removing 25–40% of near-constant features based on the interquartile range (IQR); and (c) row-wise normalization using a pseudo-reference sample constructed by averaging all control-group samples. Either unit-variance (UV) or Pareto scaling was applied to approximate a normal distribution; the choice was based exclusively on empirical observation of distributional properties.

Principal component analysis (PCA) was used to explore inherent data variation, identify potential outliers via 2D score plots, and assess analytical quality by inspecting QC sample clustering. Partial least squares-discriminant analysis (PLS-DA) was subsequently applied to identify features contributing to group separation. Model performance was evaluated by k-fold cross-validation (k = 10), leave-one-out cross-validation (LOOCV), and permutation testing (100 permutations); R^2 and Q^2 values guided parameter selection. Features with a variable importance in projection (VIP) score >1.5 were considered highly discriminant. Orthogonal PLS-DA (OPLS-DA) was additionally performed using SIMCA P+ 10.5 and EZinfo 2.0 (Umetrics, Umeå, Sweden); model quality was assessed by R^2X, R^2Y, Q^2, and 100-permutation testing.

Univariate analysis was conducted using a two-tailed, unequal-variance t-test (significance threshold: $p < 0.05$) combined with fold-change (FC) analysis (FC threshold ≥2 or ≤1/2). Group differences were visualized as a heatmap. Prior to t-testing, distributional normality was assessed using the Shapiro–Wilk, Kolmogorov–Smirnov, and Lilliefors tests (R package ‘nortest’).

Signal drift correction was performed using the QC-RLSC algorithm as implemented in the statTarget R package [S1]. The %RSD cutoff was set to 30; the QCspan parameter was set to auto (value 0), enabling the algorithm to determine the optimal smoothing span by generalized cross-validation.
